# Progressive disability but survival advantage in anti-Hu/ANNA1 paraneoplastic neurological syndromes

**DOI:** 10.1007/s00415-026-13855-5

**Published:** 2026-06-03

**Authors:** Kimberly A. DiMauro, Nicolas R. Thompson, Brittany Lapin, Nathan A. Pennell, Glen Stevens, Steven Shook, Jeffrey A. Cohen, Amy Kunchok

**Affiliations:** 1 Mellen Center for Multiple Sclerosis, Cleveland Clinic Foundation, 1950 E 89th St U Bldg, Cleveland, OH 44195 USA; 2 Department of Neurology, Cleveland Clinic Foundation, Cleveland, OH USA; 3 Department of Quantitative Health Sciences, Lerner Research Institute, Cleveland Clinic Foundation, Cleveland, OH USA; 4 Department of Hematology and Medical Oncology, Cleveland Clinic Foundation, Cleveland, OH USA; 5 Brain Tumor and Neuro-Oncology Center, Cleveland Clinic Foundation, Cleveland, OH USA

**Keywords:** Paraneoplastic, Outcomes, Disability, Encephalitis

## Abstract

**Background:**

Type 1 antineuronal nuclear or “Hu” antibody (ANNA1/Hu-IgG) is associated with paraneoplastic neurological syndromes (PNS), and most often, small cell lung carcinoma (SCLC).

**Objectives:**

To evaluate factors associated with disability and survival in ANNA1/Hu-IgG PNS and to compare the survival of patients with SCLC (with and without ANNA1/Hu-IgG PNS).

**Methods:**

This was a single-center cohort study of 45 patients with ANNA1/Hu-IgG PNS.

Kaplan–Meier methods were used to estimate time to mortality, wheelchair dependence, and modified Rankin Scale (mRS) score > 2. Cox proportional hazards models and logistic regression were used to estimate hazard ratios (HRs) and odds ratios (ORs), respectively. Secondary analyses compared patients with ANNA1/Hu-IgG PNS to a cohort with SCLC. Survival was evaluated using Cox proportional hazards models, adjusting for age, sex, cancer stage, and treatment.

**Results:**

Disability and survival were associated with clinical phenotype at onset, with limbic encephalitis having a greater hazard of mRS > 2 (HR = 6.84, 95% CI = 2.12–22.04, *P* = 0.001) and greater odds of death (HR = 2.44, 95% CI = 1.05–5.66, *P* = 0.039). Among patients with SCLC, the patients with ANNA1/Hu-IgG PNS + SCLC (*n* = 26) compared to SCLC-only (*n* = 1513) had a 41% lower hazard of death, adjusting for age, sex, stage, and cancer treatment (HR = 0.59, 95% CI = 0.37–0.96, *P* = 0.033).

**Conclusion:**

In ANNA1/Hu-IgG PNS, faster progression of disability and greater risk of mortality is associated with limbic encephalitis at onset, suggesting a potentially modifiable factor for early intervention. Patients with SCLC and ANNA1/Hu-IgG PNS survived longer than patients with SCLC without PNS, suggesting a survival advantage of paraneoplastic autoimmunity.

## Introduction

Paraneoplastic neurological syndromes (PNS) are increasingly encountered by clinicians, owing at least in part to increased awareness and availability of diagnostic antibody testing, however, little is known about the factors associated with disability and survival outcomes in this population [1, 2]. One of the most common PNS antibody biomarkers is type 1 antineuronal nuclear or “Hu” antibody (ANNA1/Hu-IgG), frequently associated with small cell lung carcinoma (SCLC) [3–6]. ANNA1/Hu-IgG PNS can present with several phenotypes, including limbic encephalitis, cerebellar degeneration, dysautonomia, myelopathy, myopathy and neuropathy, among others [7–9]. ANNA1/Hu-IgG PNS are typically found in older patients, often in the sixth decade [10–12].

While there are case reports and cohort studies detailing clinical phenotypes and associated cancers, there are few studies reporting long-term disability outcomes of this patient population [13, 14]. In a cohort study of 16 ANNA1/Hu-IgG PNS with cerebellar degeneration, 8 (50%) had clinical deterioration defined as a Rankin score change of ≥ 1 point after diagnosis. Older age and a higher level of disability were associated with worse functional outcome, whereas tumor treatment and immunosuppressive therapy showed no difference [15]. Prognosis overall for this patient population has traditionally been thought to be poor, but a full understanding of factors associated with morbidity and mortality is lacking [16].

Survival for patients with SCLC is poor, estimated at 7.8 months (95% confidence interval [CI] = 7.6–8.0 months) from diagnosis for all stages; for those with stage I–III, survival was 14.1 months (95% CI = 13.3–15.0) in recent studies, while stage IV survived 6.3 months (95% CI = 6.0–6.6) [17]. It remains unclear, however, whether survival in SCLC is different in patients with PNS [18–20]. It has been hypothesized that paraneoplastic autoimmunity may be associated with a greater anti-tumor effect, limited tumor growth and better survival for patients with PNS [21]. It also is hypothesized that the clinical manifestation of PNS leads to earlier cancer diagnoses. A cohort of ANNA1/Hu-IgG PNS patients found that a large percentage had ANNA1/Hu-IgG-specific T and B cells within the tumor, and appeared to improve the natural history of the disease [22]. A case series described the spontaneous regression of SCLC in patients with paraneoplastic antibodies [23]. Additionally, animal model studies have suggested that paraneoplastic autoimmunity may be associated with an anti-tumor effect. Mice immunized with ANNA1/Hu-IgG showed tumor growth inhibition of neuroblastoma (51% reduction in volume; *P* = 0.0012), with 14% of them with complete tumor rejection, in comparison to controls [24]. Retrospective cohort studies have reported heterogenous survival outcomes including similar survival in ANNA1/Hu-IgG PNS compared to SCLC without PNS, and increased survival attributed to lead-time bias [25, 26]. Given the discrepancies and limited data that currently exist, further studies are needed to better understand the potential impact of paraneoplastic autoimmunity upon cancer survival [14, 18].

This study aimed to evaluate the association of clinical factors upon disability outcomes and survival in ANNA1/Hu-IgG PNS, and the impact of paraneoplastic autoimmunity on cancer survival in patients with ANNA1/Hu-IgG PNS and SCLC.

## Methods

### Standard protocol approvals, registrations, and patient consents

The study protocol was approved by the Cleveland Clinic Institutional Review Board (IRB). The IRB waived the need for individual patient consent for this study. The Strengthening the Reporting of Observational Studies in Epidemiology (STROBE) reporting guidelines were used.

### Study design and population

This is a single-center, retrospective, observational cohort study of patients with ANNA1/Hu-IgG PNS evaluated at Cleveland Clinic from January 2002 to July 2023. Patients were identified through the Cleveland Clinic autoimmune clinic registry and the electronic medical record search. Inclusion criteria included demonstration of ANNA1/Hu-IgG in serum and/or cerebrospinal fluid (CSF). The testing was completed at Mayo Clinic (60%) by indirect immunofluorescence and western blot, and at Athena Diagnostics (40%) by immunoassay. All patients included were diagnosed by the treating clinician as having PNS. Further, these diagnoses were reviewed by KD and AK to determine that they met diagnostic criteria for PNS [27]. A control group of SCLC (histologically confirmed) was identified from the Cleveland Clinic Cancer Center database and was screened for clinical diagnoses of ANNA1/Hu-IgG PNS.

### Clinical factors

Clinical factors included clinical phenotype, cancer diagnosis, and treatments. These were chosen as independent variables for the models based on clinical association with outcomes identified in previous reports [5, 6]. The description of the cohort included inflammatory CSF which was defined as either CSF pleocytosis (> 5 cells/uL), elevated protein (> 45 mg/dL), and/or presence of CSF-specific oligoclonal bands (OCBs). The description of an abnormal brain MRI was defined as either temporal lobe T2/FLAIR hyperintensities, cerebellar atrophy, mesial temporal sclerosis, or abnormal enhancement and excluded metastases.

### Outcome measures

Outcome measures included mRS defined as a score of 0 having no disability, a score of 1 having nonsignificant disability, a score of 2 with slight disability, but able to live independently, a score of 3 with moderate disability causing dependency, but not loss of ambulation without assistance of another person, a score of 4 as loss of ambulation without assistance of another person and/or loss of ability to perform self-care, a score of 5 as bedridden needing continuous care [28]. A sensitivity analysis of patients who had mRS 0–2 at baseline was conducted to evaluate the time from symptom onset to mRS > 2 and mRS > 2 at last follow-up.

Development of wheelchair dependence at last follow-up was a binary outcome (excluding patients in a wheelchair at baseline). Time from symptom onset to the first recorded date of wheelchair use within the medical record was also examined. Death date was determined from the medical records and the oncology database.

### Statistical analysis

Descriptive statistics (mean (SD), median, (interquartile range, IQR)) summarized patient and clinical characteristics of the cohort. Kaplan–Meier curves for time to mortality, time to wheelchair dependence (for patients who were not wheelchair dependent at baseline), and time to mRS > 2 (for patients who had mRS ≤ 2 at baseline) were constructed for the entire cohort, and stratified by neurological phenotypes, immunosuppressive therapy use, and presence of cancer. The Log-rank test assessed whether each time-to-event outcome differed by group.

To determine the impact of clinical factors upon disability and survival outcomes, single-predictor Cox proportional hazard models were utilized. Hazard ratios along with 95% confidence intervals were computed. We examined which clinical factors were associated with the last mRS > 2 using Firth’s logistic regression.

For comparison of survival among SCLC patients (ANNA1/Hu-IgG PNS + SCLC versus SCLC-only), Kaplan–Meier curves for each of the 2 groups were constructed. Time from date of SCLC diagnosis until death was set as the dependent variable. Surviving patients were right-censored at their last follow-up visit. A log-rank test was used to assess survival differences. Cox proportional hazard models were fitted to obtain hazard ratios and 95% confidence intervals. A secondary analysis with adjustment for age, sex, cancer stage, and cancer treatment was completed. Computations were performed in R, version 4.3.1. All tests were two-sided and p-values < 0.05 were considered statistically significant.

### Data availability

Anonymized data not published within this article can be made available by reasonable request from any qualified investigator subject to approval by the Cleveland Clinic Institutional Review Board.

## Results

### Study population

Among 45 patients with ANNA1/Hu-IgG PNS, the mean age was 63 years (SD = 12.9) and 73% were female. A full descriptive table is provided in Table 1. A total of 1,539 patients with SCLC were evaluated for survival. This included 26 with ANNA1/Hu-IgG PNS + SCLC and 1513 with SCLC-only.

**Table 1:** Descriptive characteristics of ANNA1/Hu-IgG paraneoplastic neurological syndrome cohort

*N*	45
Age, mean (SD)	62.8 (12.9)
Female	33/45 (73.3%)
Smoking history	40/45 (88.9%)
*Clinical features*	
Lambert-Eaton Myasthenic syndrome*	3/45 (6.7%)
Neuropathy*	22/45 (48.9%)
Limbic encephalitis*	11/45 (24.4%)
Cerebellar ataxia*	10/45 (22.2%)
Gastrointestinal dysmotility*	3/45 (6.7%)
Myelopathy	2/45 (4.4%)
Myoclonus	1/45 (2.2%)
*Neurological system involved at onset*	
Central	24/45 (53.3%)
Peripheral	21/45 (46.7%)
*Co-existing Antibodies*	15/37 (40.5%)
ANNA2/Ri	1/37 (2.7%)
CRMP5/CV2	5/37 (13.5%)
mAChR	1/37 (2.7%)
PCA2	2/37 (5.4%)
P/Q-type voltage-gated calcium channel	8/37 (21.6%)
Cancer diagnosis following PNS onset	23/35 (65.7%)
Cancer diagnosis prior to PNS onset	12/35 (34.3%)
*Cancer type*	
SCLC	26/35 (74.3%)
Non-SCLC	4/35 (11.4%)
Other (non-lung cancer)	5/35 (14.3%)
Brain metastases	2/44 (4.5%)
Inflammatory CSF	20/28 (71.4%)
Immune check point inhibitor use	1/45 (53.3%)
Immunosuppressive therapy	24/45 (53.3%)
Follow-up time (years), median (IQR)	1.31 (0.71, 4.48)

*SD* standard deviation, *PNS* paraneoplastic neurologic syndrome, *SCLC* small cell lung cancer, *IQR* interquartile range

* representation of multiple phenotypes

### Clinical, serological and radiographical phenotypes of ANNA1/Hu-IgG PNS

**Fig. 1:**
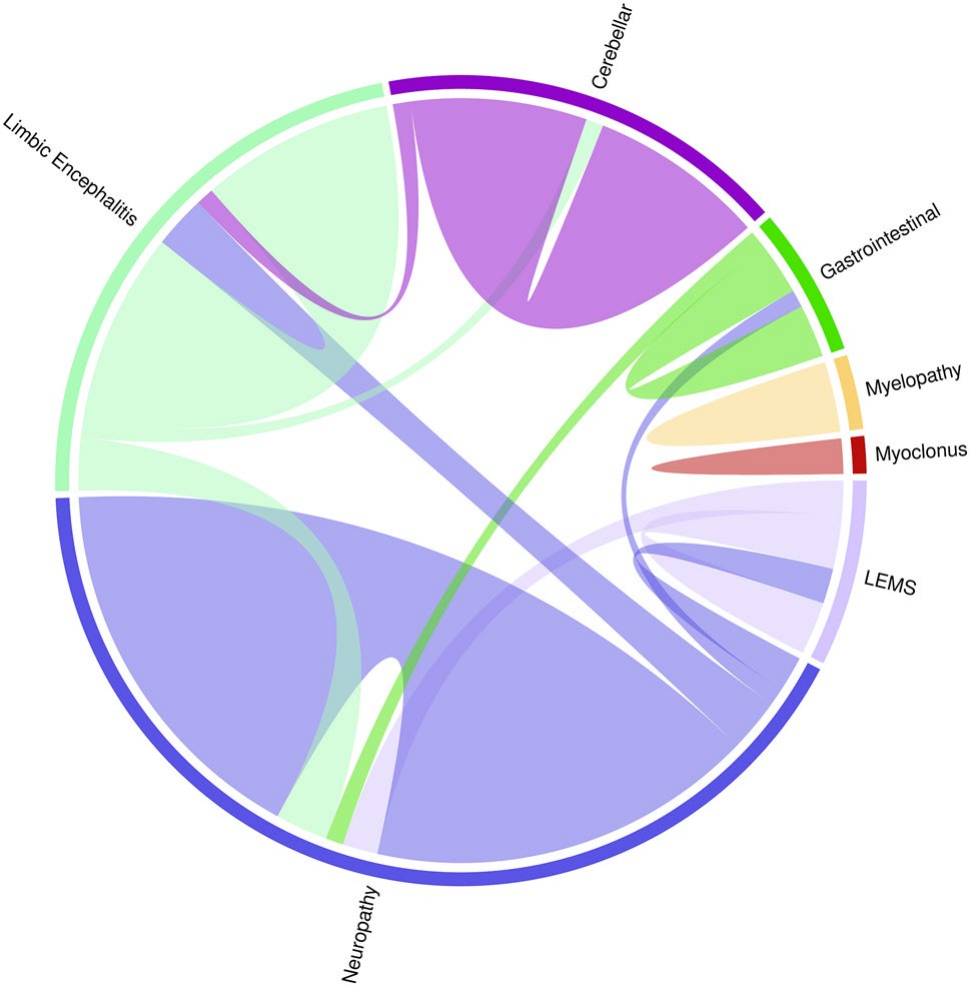
Chord diagram depicting overlapping ANNA1/Hu-IgG paraneoplastic neurological syndrome phenotypes

Initial neurological presentations of PNS were central (24, [53%]) and peripheral (21, [47%]). Central involvement included limbic encephalitis (11, [24%]), cerebellar ataxia (10, [22%]), myelopathy (2, [4%]), myoclonus (1, [2%]). Peripheral involvement included: neuropathy (22, [49%]; 3 were sensory ganglionopathy), Lambert-Eaton myasthenic syndrome (3, [7%]), gastrointestinal dysmotility (including pseudo-obstruction) (3, [7%]). There were 2 patients additionally with sensorineural hearing loss. Overlapping phenotypes were seen in 7 (16%) and were depicted in Fig. 1. Neurologic phenotypes are summarized in Table 1.

Co-existing antibodies were present in serum or CSF in 15 (41%) of 37 patients with other antibody testing available, with multiple antibodies in 2 patients. Co-existing antibodies included VGCC P/Q-type voltage-gated calcium channel (8), CRMP5/CV2 (5), PCA2 (2), mAChR (1), and ANNA2/Ri (1). Inflammatory CSF findings were present in 20 of 28 (71%) patients with CSF analysis. Brain MRI demonstrated abnormal features of PNS in 13/44 (30%) patients with MRI. This included 9 patients with T2/FLAIR hyperintensities, of whom 6 had mesial temporal lobe changes. Four patients had cerebellar atrophy. Two patients with abnormal MRI had abnormal contrast-enhancement, one with enhancement in the frontal subcortex and the other with enhancement in the uncus. Brain metastases were present in 2/44 (4.5%) patients of the total patient cohort with MRI available.

### Immunosuppressive therapies

There were 24 (53%) who were treated with immunosuppressive therapies including: IV/oral steroids (22), IVIG (19), plasmapheresis (6), and cyclophosphamide (5); the majority (18, [75%]) received more than one therapy. Clinical improvement post-immunosuppression was reported by the treating doctor in 11 (46%). There were 21 who were not treated with immunosuppressive therapies due to various factors including: concurrent cancer treatment, prognosis, comorbidities, and patient or clinician preference. Nearly half of the patients not treated with immunosuppression (10; 48%) had peripheral neuropathy or gastrointestinal dysmotility.

### Oncological diagnoses and treatments

Cancer was identified in 35 patients (78%). Twenty-three (66%) were diagnosed with cancer following their PNS onset, and 12 (34%) were diagnosed with cancer before their PNS onset. For the 23 patients who had cancer diagnosed after their PNS, the median time from PNS onset to cancer diagnosis was 8.6 months (IQR = 3.9–16.9). Four (9%) of the total cohort of 45 patients were diagnosed with cancer > 2 years after the onset of their PNS.

A history of smoking was reported in 40 (89%) patients. SCLC was the most common associated cancer (26, [74%]). Other cancers included non-small cell lung cancer (4, [11%]), colon (2, [6%]), gallbladder (1, [3%]), prostate (1, [3%]), and neuroendocrine (1, [3%]). Twenty-five (71%) patients were treated with chemotherapy, either alone or in combination with radiation and/or surgical resection. One patient was treated with the immune checkpoint inhibitor (ICI) atezolizumab, without pre-existing symptoms or diagnosis of PNS and subsequently developed de novo PNS approximately 2 months after ICI initiation.

### Clinical factors associated with disability outcomes

**Fig. 2:**
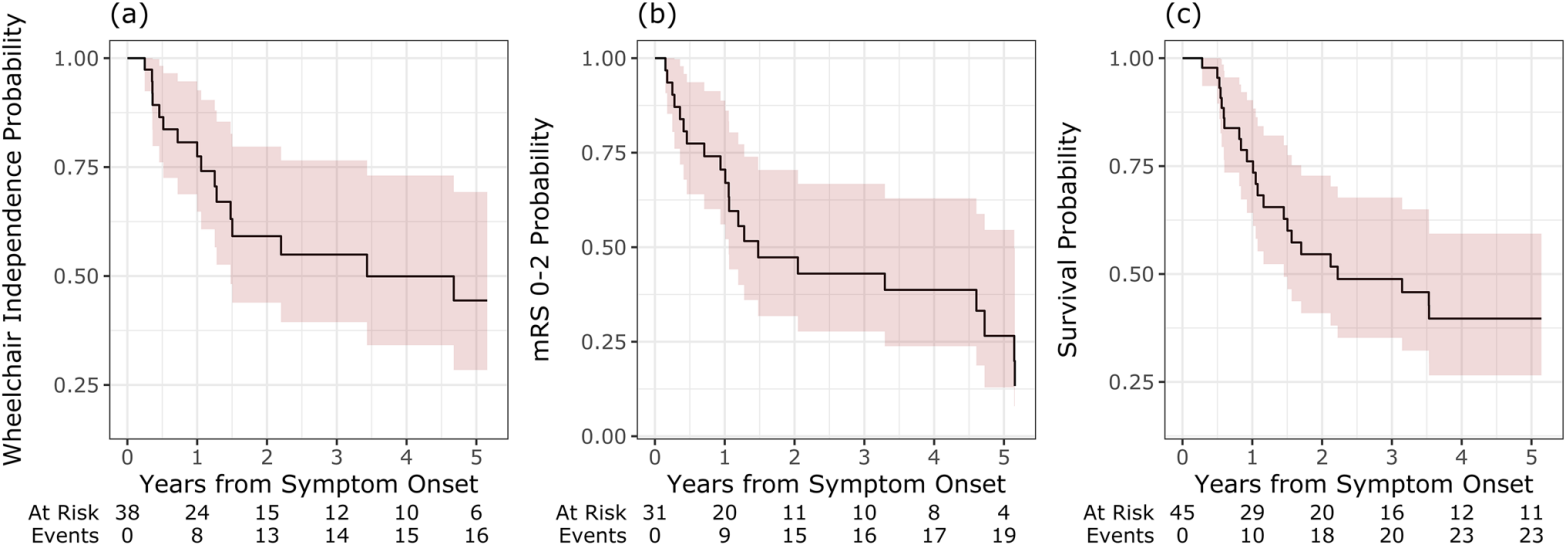
**a**–**c** Kaplan Meier curves for disability outcomes in the ANNA1/Hu-IgG paraneoplastic neurological syndrome cohort for (**a**) time to wheelchair dependence (for patients who were not wheelchair dependent at baseline), (**b**) time to modified Rankin Scale (mRS) score > 2 (for patients whose baseline was mRS 0–2), and (**c**) time to mortality

The median follow-up for the cohort was 1.31 years (IQR = 0.71–4.48). At baseline, the median mRS was 2 (IQR = 1–3), 14/45 (31%) patients had mRS > 2 and there were 7/45 (16%) patients who were wheelchair dependent. The proportion of patients who were wheelchair dependent was 41% at 2 years post-symptom-onset and 56% at 5 years post-symptom-onset (Fig. 2a). The proportion of patients who had mRS > 2 was 53% at 2 years post-symptom-onset and 74% at 5 years post-symptom-onset (Fig. 2b).

**Fig. 3:**
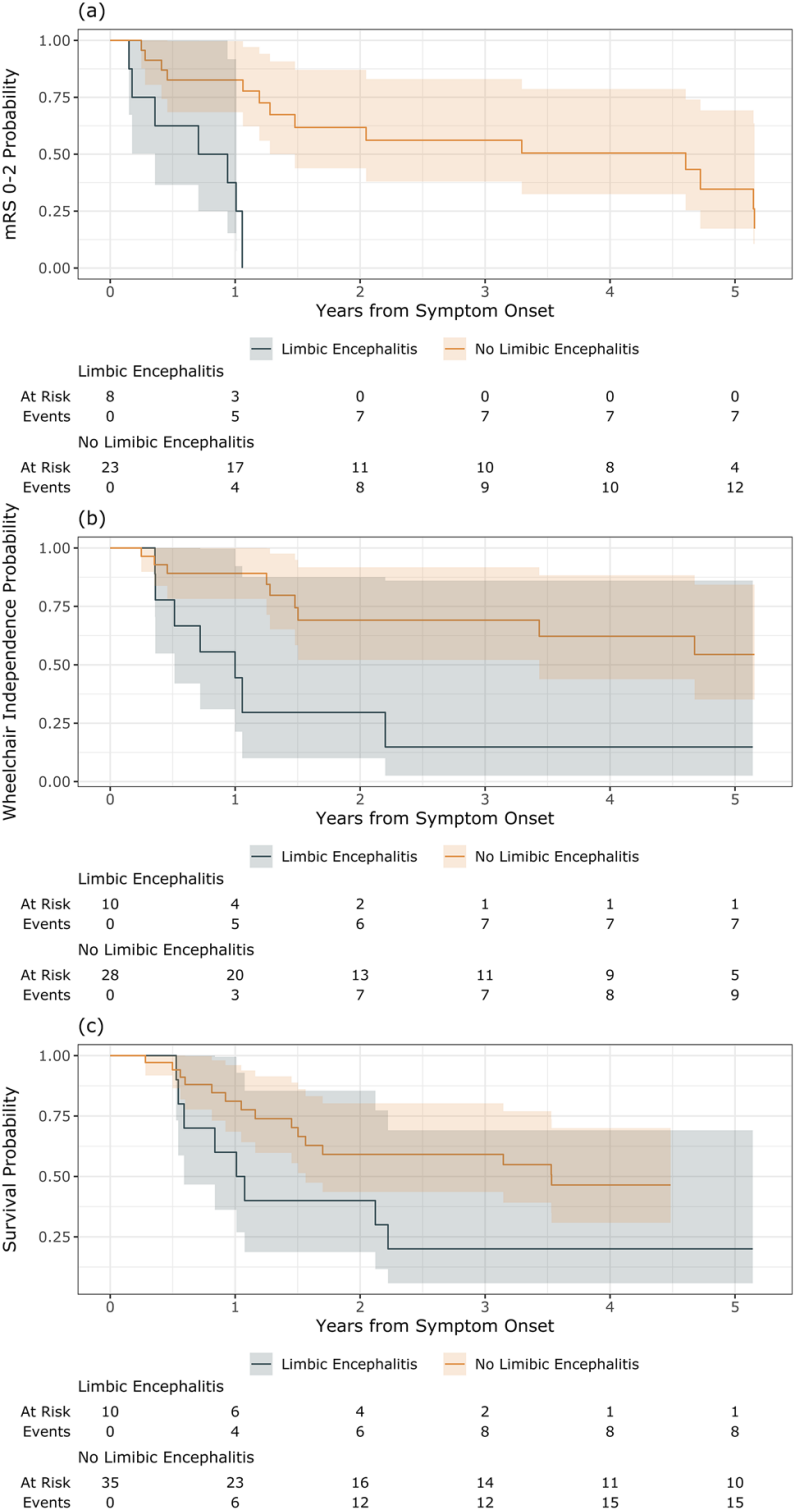
**a**–**c** Kaplan Meier curves for (**a**) time to modified Rankin Scale (mRS) score > 2, stratified by limbic encephalitis (analysis restricted to those with baseline mRS 0–2), (**b**) time to wheelchair dependence, stratified by limbic encephalitis (analysis restricted to those not wheelchair dependent at baseline), and (**c**) time to mortality

Patients with limbic encephalitis had a greater hazard of reaching mRS > 2 than patients without limbic encephalitis (HR = 6.84, 95% CI 2.12–22.04, *P* = 0.001) (Fig. 3a; Table 2). Patients with limbic encephalitis had a greater hazard of reaching wheelchair dependence than patients who did not have limbic encephalitis (HR = 3.31, 95% CI 1.20–9.12, *P* = 0.021) (Fig. 3b; Table 2). No other clinical or treatment factors were associated with disability (Table 2).

**Table 2:** Cox proportional hazard models for survival, time to wheelchair dependence (for patients who were not wheelchair dependent at baseline), time to mRS > 2 (for patients whose baseline mRS was 0–2), and a logistic regression model for the last mRS

	Survival (Cox proportional hazard)			Wheelchair dependence (Cox proportional hazard)			Time to mRS > 2 (Cox proportional hazard)			Last mRS > 2 (Logistic regression)		
	*N*	Hazard ratio (95% CI)	*P*-value	*N*	Hazard ratio (95% CI)	*P*-value	*N*	Hazard ratio (95% CI)	*P*-value	*N*	Odds ratio (95% CI)	*P*-value
Cancer	35/45	1.19 (0.45, 3.14)	0.731	32/38	1.16 (0.33, 4.14)	0.816	25/31	2.28 (0.64, 8.06)	0.202	25/31	1.30 (0.11, 9.27)	0.805
SCLC^a^	26/35	1.30 (0.48, 3.57)	0.607	24/32	1.55 (0.43, 5.58)	0.503	17/25	1.97 (0.64, 6.08)	0.239	17/25	2.38 (0.31, 18.94)	0.392
Lung cancer	30/35	1.83 (0.42, 7.95)	0.418	27/32	1.08 (0.24, 4.85)	0.919	20/25	1.68 (0.38, 7.47)	0.496	20/25	5.29 (0.63, 48.18)	0.122
Immunosuppressive therapies	24/45	0.95 (0.44, 2.04)	0.896	21/38	1.62 (0.61, 4.35)	0.335	17/31	1.62 (0.68, 3.89)	0.279	17/31	1.89 (0.31, 13.03)	0.484
Corticosteroid use	22/45	0.80 (0.37, 1.72)	0.572	19/38	1.58 (0.61, 4.09)	0.344	15/31	1.61 (0.69, 3.79)	0.273	15/31	1.40 (0.23, 9.62)	0.711
Corticosteroid ≥ 1 month	11/45	0.54 (0.22, 1.37)	0.195	11/38	1.16 (0.44, 3.02)	0.766	9/31	0.98 (0.41, 2.38)	0.967	9/31	1.38 (0.21, 15.32)	0.751
IVIg	19/45	0.85 (0.40, 1.84)	0.687	18/38	1.28 (0.50, 3.27)	0.601	14/31	1.39 (0.60, 3.25)	0.443	14/31	3.00 (0.47, 32.93)	0.255
PNS (vs. CNS) at onset	21/45	0.83 (0.39, 1.78)	0.629	17/38	0.64 (0.25, 1.66)	0.362	15/31	0.52 (0.22, 1.23)	0.137	15/31	1.40 (0.23, 9.62)	0.711
Isolated neuropathy	17/45	0.98 (0.45, 2.12)	0.959	13/38	0.50 (0.18, 1.42)	0.196	12/31	0.48 (0.20, 1.19)	0.113	12/31	2.23 (0.35, 24.46)	0.414
Ataxia	10/45	0.68 (0.23, 1.97)	0.479	8/38	0.73 (0.21, 2.54)	0.621	6/31	0.70 (0.23, 2.10)	0.528	6/31	0.28 (0.04, 2.09)	0.202
Limbic encephalitis	11/45	2.44 (1.05, 5.66)	0.039	11/38	3.31 (1.20, 9.12)	0.021	9/31	6.84 (2.12, 22.04)	0.001	9/31	5.97 (0.57, 815.85)	0.155

^a^compared to patients with other cancers

*mRS* modified Rankin Scale, *CI* confidence interval, *PNS* peripheral nervous system, *CNS* central nervous system, *SCLC* small cell lung cancer

### Clinical factors associated with survival outcomes

The proportion of patients who died was 45% at 2 years post-symptom onset and 60% at 5 years post-symptom onset (Fig. 2c). For the full cohort of 45 patients, median survival time was 26.7 months (95% CI = 17.4–144.0).

Patients with limbic encephalitis had a greater hazard of death than patients who did not have limbic encephalitis (HR = 2.44, 95% CI 1.05–5.66, *P* = 0.039) (Fig. 3c; Table 2). The cause of death was due to cancer or cancer-related complications in 17 [63%], unrelated infection in 1 [4%], and unknown in 9 [33%]. No deaths were observed from PNS. No treatment factors were found to be associated with survival (Table 2).

### Differences in survival of SCLC with and without ANNA1/Hu-IgG PNS

To evaluate the impact of paraneoplastic autoimmunity upon survival outcomes, we examined a subset of patients with ANNA1/Hu-IgG PNS and SCLC and compared this group to SCLC patients without ANNA1/Hu-IgG PNS. Full descriptive statistics between these two groups are provided in Table 3. For the 26 ANNA1/Hu-IgG PNS + SCLC patients, median survival time from SCLC diagnosis was 19.2 months (95% CI = 7.8–Not Reached). We were unable to calculate the upper bound for median survival in the 26 SCLC patients because of sample size issues for estimating median survival time. Among the 1513 SCLC-only patients, median survival was 12.5 months (95% CI = 11.8–13.4).

**Table 3:** Comparative descriptive characteristics of small-cell lung cancer-only and ANNA1/Hu-IgG paraneoplastic neurological syndrome + small-cell lung cancer cohorts

	All patients	SCLC-only	ANNA1/Hu-IgG + SCLC	*P*-value
*N*	1539	1513	26	
Age, mean (SD)	65.9 (9.9)	65.9 (9.9)	64.6 (8.8)	0.456
Female	795/1539 (51.7%)	774/1513 (51.2%)	21/26 (80.8%)	0.003
Smoking history	1409/1432 (98.4%)	1385/1406 (98.5%)	24/26 (92.3%)	0.064
*Stage*				
Limited	543/1352 (40.2%)	528/1326 (39.8%)	15/26 (57.7%)	0.072
Extensive	809/1352 (59.8%)	798/1326 (60.2%)	11/26 (42.3%)	
*Brain metastases*				
Yes	240/1179 (20.4%)	239/1153 (20.7%)	1/26 (3.8%)	0.044
No	939/1179 (79.6%)	914/1153 (79.3%)	25/26 (96.2%)	
*Immune checkpoint inhibitor*				
Yes	229/1496 (15.3%)	228/1470 (15.5%)	1/26 (3.8%)	0.163
*Cancer treatment*				
None	215/1539 (14.0%)	211/1513 (13.9%)	4/26 (15.4%)	0.776
Chemotherapy	1324/1539 (86.0%)	1302/1513 (86.1%)	22/26 (84.6%)	

*SD* standard deviation, *SCLC* small cell lung cancer

**Fig. 4:**
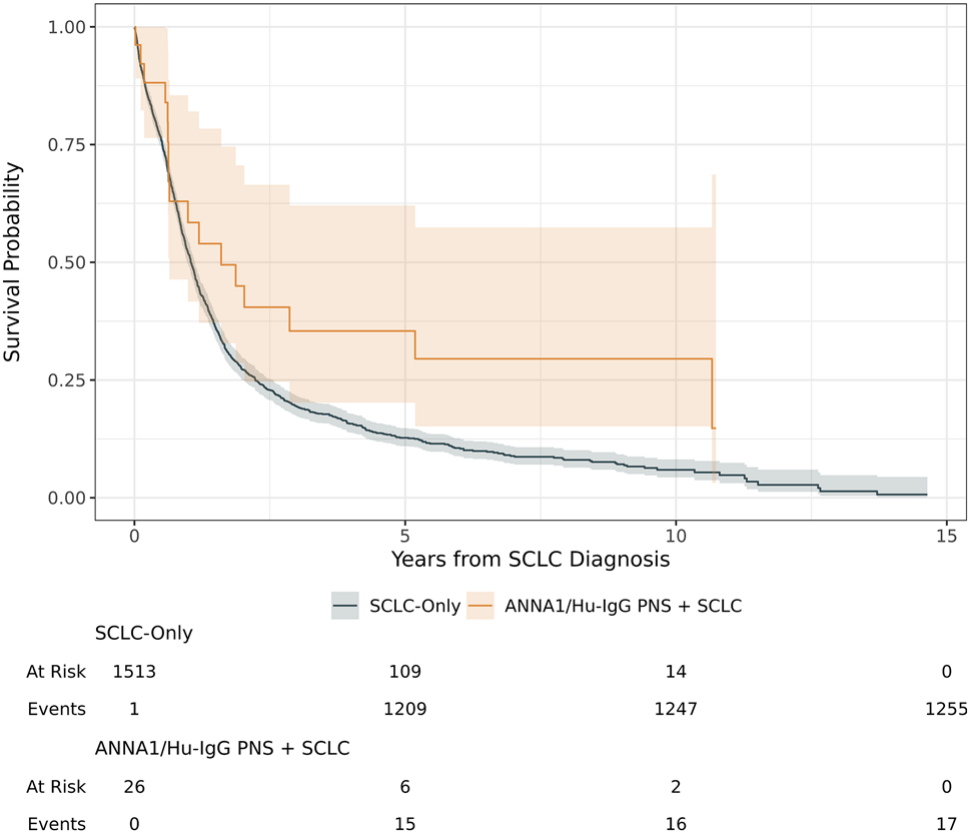
Comparison of probability of survival for small-cell lung cancer (SCLC)-only group and SCLC + ANNA1/Hu-IgG paraneoplastic neurological syndrome

The SCLC-only patients had significantly shorter survival (log-rank test *P* = 0.030) than ANNA1/Hu-IgG PNS + SCLC patients (Fig. 4). Survival at 2, 5, and 10 years was 27%, 13%, and 6% for SCLC patients and was 53%, 34%, and 29% for ANNA1/Hu-IgG PNS + SCLC patients. Cox proportional-hazards model adjusting for age, sex, cancer stage, and cancer treatment revealed that ANNA1/Hu-IgG PNS + SCLC patients had a 41% lower hazard of death (HR = 0.59, 95% CI 0.37–0.96, *P* = 0.033) when compared to SCLC-only patients (Table 4).

**Table 4:** Cox proportional hazard models with various covariate adjustment comparing survival between small cell lung cancer (SCLC)-only (reference group) and SCLC + ANNA1/Hu-IgG paraneoplastic neurological syndrome patients

Model Adjustment	HR (95% CI)	*P*-value
None	0.59 (0.37, 0.95)	0.032
Age, Sex, Stage	0.58 (0.36, 0.93)	0.025
Age, Sex, Stage, Cancer Treatment	0.59 (0.37, 0.96)	0.033

*HR* hazard ratio, *CI* confidence interval

## Discussion

This study identified that despite patients with ANNA1/Hu-IgG PNS developing disability, they paradoxically had improved survival compared to matched SCLC controls, controlled for cancer stage (in addition to age, sex and cancer treatment). The overall observation of improved survival is important, as it raises questions regarding whether paraneoplastic autoimmunity can be beneficial in tumor control. This study also examined clinical outcomes and found that disability accrual and mortality were greater in ANNA1/Hu-IgG PNS with limbic encephalitis, as opposed to other clinical manifestations, suggesting that aggressive and early targeting of this phenotype may act as a potentially modifiable factor to reduce disability.

Although > 50% of the cohort had died at 5 years, patients with ANNA1/Hu-IgG PNS + SCLC survived longer than SCLC patients without PNS. This finding is similar to a prior study that found a higher probability of survival at 30 months in ANNA1/Hu-IgG PNS + SCLC patients compared to SCLC alone [14]. Further, our ANNA1/Hu-IgG PNS + SCLC had a median survival time of 19.2 months, compared to the median survival for SCLC-only of 7.2 months [17]. In our SCLC-only cohort, median survival was 12.5 months.

One potential cause for increased survival in the ANNA1/Hu-IgG PNS + SCLC group compared to SCLC alone is that paraneoplastic autoimmunity represents augmented-tumor immunity. Paraneoplastic autoimmunity is T-cell mediated and this has been demonstrated in pathology studies in which cytotoxic CD8 + T cells were found in both neoplastic lymph node tissue and dorsal root ganglia in patients with PNS [29]. Other pathology studies have demonstrated a T cell inflammatory infiltrate in the tumor tissue of patients with ANNA1/Hu-IgG PNS [30, 31]. Additionally, abundant T cell infiltration also has been found in the tumor tissue for patients with other PNS, such as PCA1/Yo-IgG cerebellar degeneration [32]. Together with the observation of onconeural antigen-specific CD8 + T cells in the blood and/or tissue of ANNA1/Hu-IgG PNS patients, this may suggest that the patients with PNS experience increased anti-tumor immunity [22, 29–31].

The hypothesis that paraneoplastic autoimmunity may increase survival due to augmented-tumor effect is supported by the greater proportion of PNS patients with early-stage conditions [19, 22, 33]. Further, it has been demonstrated in several early and late-stage cancers that ICI improve survival by augmenting the anti-tumor immune response through the blockade of inhibitory regulatory checkpoints [34–36]. This results in increasing T cell activation and reversal of T cell exhaustion which increases the anti-tumor effect, thereby increasing survival [37, 38]. Therefore, similar to ICI, it is possible that paraneoplastic autoimmunity increases T cell activation and reverses T cell exhaustion, resulting in better control of the tumor and prolonged survival.

An additional reason for the prolonged survival seen is that some PNS may have earlier cancer detection (lead-time bias). Some studies have suggested that the presence of ANNA1/Hu-IgG PNS is associated with limited stage disease and thus the possibility of lead-time bias has been raised [18, 22, 25]. One study, however, found that ANNA1/Hu-IgG presence independent from a PNS had a potential role of predicting SCLC survival [19]. Another study, even when accounting for lead-time bias, still found a survival advantage in comparison to SCLC [39]. The variations in results may argue that both lead-time bias and anti-tumor immunity play a role in this observed survival advantage.

Other observations from our study that align with retrospective PNS studies were that 9% of patients were diagnosed with cancer > 2 years after onset of PNS, re-enforcing the need for ongoing oncologic surveillance if cancer is not initially identified [6].

Limitations of this study include a small sample size limiting statistical power and a retrospective design due to the rarity of ANNA1/Hu-IgG PNS. The lower number of identified sensory neuronopathies among the neuropathies in our cohort compared to known larger cohort studies may reflect insufficient or suboptimal nerve conduction study data and thus impact phenotypic characterization. Treatments were not associated with disability, but we were unable to assess this comprehensively due to power, heterogeneity of overlapping treatments, and potential indication bias.

This study demonstrates that faster progression of disability in ANNA1/Hu-IgG PNS is associated with limbic encephalitis, suggesting a potentially modifiable outcome. Furthermore, the results showed that patients with ANNA1/Hu-IgG PNS + SCLC survived longer than SCLC alone, suggesting that despite accruing neurological disability, ANNA1/Hu-IgG paraneoplastic autoimmunity may confer a survival advantage.
